# Molecular characterization of three Rhesus glycoproteins from the gills of the African lungfish, *Protopterus annectens*, and effects of aestivation on their mRNA expression levels and protein abundance

**DOI:** 10.1371/journal.pone.0185814

**Published:** 2017-10-26

**Authors:** You R. Chng, Jasmine L. Y. Ong, Biyun Ching, Xiu L. Chen, Kum C. Hiong, Wai P. Wong, Shit F. Chew, Siew H. Lam, Yuen K. Ip

**Affiliations:** 1 Department of Biological Sciences, National University of Singapore, Singapore; 2 Natural Sciences and Science Education, National Institute of Education, Nanyang Technological University, Singapore; 3 NUS Environmental Research Institute, National University of Singapore, Singapore; Universidade de Brasilia, BRAZIL

## Abstract

African lungfishes are ammonotelic in water. They can aestivate for long periods on land during drought. During aestivation, the gills are covered with dried mucus and ammonia excretion ceases. In fishes, ammonia excretion through the gills involves Rhesus glycoproteins (RhGP/Rhgp). This study aimed to obtain the complete cDNA coding sequences of *rhgp* from the gills of *Protopterus annectens*, and to determine their branchial mRNA and protein expression levels during the induction, maintenance and arousal phases of aestivation. Three isoforms of *rhgp* (*rhag*, *rhbg* and *rhcg*) were obtained in the gills of *P*. *annectens*. Their complete cDNA coding sequences ranged between 1311 and 1398 bp, coding for 436 to 465 amino acids with estimated molecular masses between 46.8 and 50.9 kDa. Dendrogramic analyses indicated that Rhag was grouped closer to fishes, while Rhbg and Rhcg were grouped closer to tetrapods. During the induction phase, the protein abundance of Rhag, but not its transcript level, was down-regulated in the gills, suggesting that there could be a decrease in the release of ammonia from the erythrocytes to the plasma. Furthermore, the branchial transcript levels of *rhbg* and *rhcg* decreased significantly, in preparation for the subsequent shutdown of gill functions. During the maintenance phase, the branchial expression levels of *rhag*/Rhag, *rhbg*/Rhbg and *rhcg*/Rhcg decreased significantly, indicating that their transcription and translation were down-regulated. This could be part of an overall mechanism to shut down branchial functions and save metabolic energy used for transcription and translation. It could also be regarded as an adaptive response to stop ammonia excretion. During the arousal phase, it is essential for the lungfish to regain the ability to excrete ammonia. Indeed, the protein abundance of Rhag, Rhbg and Rhcg recovered to the corresponding control levels after 1 day or 3 days of recovery from 6 months of aestivation.

## Introduction

Lungfishes are an archaic group of Sarcopterygian fishes that possess lung opening off the ventral side of the oesophagus. To date, there are six species of extant lungfish worldwide, including *Protopterus annectens*, *P*. *aethiopicus*, *P*. *amphibicus* and *P*. *dolloi* from Africa. African lungfishes are obligate air-breathers living in fresh water. During drought, they can aestivate in subterranean mud cocoon for up to ~4 years [1–3]. In the laboratory, African lungfishes can be induced to aestivate in completely dried mucus cocoons in plastic boxes [4–6].

Aestivation involves corporal torpor at high environmental temperature without food or water intake for an extended period [1,3]. There are three phases of aestivation: induction, maintenance, and arousal. During the induction phase, the aestivating lungfish detects environmental cues and turns them into internal signals that will initiate the necessary changes to prepare for the maintenance phase. Within 6–8 days, it hyperventilates and secretes mucus which turns into a dry cocoon. During the maintenance phase, the lungfish is completely encased in a cocoon with no feeding and movement. During this period, the lungfish has to prevent cell death, preserve biological structures and sustain a slow rate of waste production to avoid pollution of the internal environment. The lungfish can be aroused from aestivation by the addition of water. Upon arousal, it has to excrete the accumulated waste products and feed for repair and growth. Feeding begins approximately 7–10 days after arousal, and the fish grows and develops as normal thereafter.

Despite being ureogenic [7–9], African lungfishes are ammonotelic in water [10]. They possess a full complement of ornithine-urea cycle enzymes, including glutamine synthetase, carbamoyl phosphate synthetase III (Cps III), ornithine transcarbamylase, argininosuccinate synthase (Ass), argininosuccinate lyase (Asl) and arginase in their livers [6,11,12]. Exposure to terrestrial conditions leads to decreases in ammonia and urea excretion in the African lungfishes, a phenomenon attributable presumably to a lack of water to flush the branchial and cutaneous surfaces [10]. However, ammonia is not just passively retained in the lungfish during emersion; it is converted into urea in the liver and urea is accumulated in the body instead. It was previously reported that aerial exposure led to an increase in the hepatic ornithine-urea cycle capacity, with significant increases in the activities of Cps III (3.8-fold), Ass + Asl (1.8-fold) and glutamine synthetase (2.2-fold) [4]. Furthermore, there were decreases in the rate of ammonia production and increases in the rate of urea synthesis in *P*. *dolloi* during 6 days of aerial exposure or 40 days of aestivation in a dried mucus cocoon [4,10]. It has been postulated that the urea accumulated during the induction phase can act as an internal cue for aestivation [13]. Hence, it is possible that African lungfishes down-regulate the transport mechanisms of ammonia and urea in its body surfaces, especially the gills, during the induction phase. During the maintenance phase, urea accumulation continues, indicating the continuous but impeded mobilization of endogenous proteins/amino acids [4,14,15]. The gills become physiologically nonfunctional as they are covered with a thick layer of dried mucus [16], but whether there is a shutdown of branchial function through down-regulation of transporters/channels, including those involved in ammonia and urea excretion, is uncertain. Upon arousal from aestivation, the lungfish rapidly excretes the accumulated urea [1] and revert back to become ammonotelic thereafter. Hence, there must be a restoration of expression of nitrogenous waste transporters in the gills during the arousal phase.

Rhesus glycoproteins (RhGP/Rhgp) are members of the solute transporter family SLC42 that play an important role in transmembrane ammonia transport [17,18]. There are four types of RhGP, three of which, namely Rh-associated glycoprotein (RhAG), Rh family B glycoprotein (RhBG) and Rh family C glycoprotein (RhCG), are glycosylated. The fourth type, Rh30, lacks glycosylation and is found to be associated with RhAG in erythrocytic membranes. For most ammonotelic fishes, ammonia is mainly excreted across the branchial epithelium as NH_3_ down a favourable blood-to-water diffusion gradient [19–21]. Wright and Wood [22] proposed a model for ammonia excretion in freshwater fishes and its variable connection to Na^+^ uptake and acid excretion. In this model, Rhag facilitates NH_3_ flux out of the erythrocyte, Rhbg moves it across the basolateral membrane of the branchial ionocyte, and an apical “Na^+^/NH_4_^+^ exchange complex” consisting of several membrane transporters (Rhcg, vacuolar-type H^+^-ATPase, Na^+^/H^+^ exchanger, Na^+^ channel) working together as a metabolon to provide an acid trapping mechanism for apical excretion. This model incorporates the premise that Rhgp function as ammonia channels, binding NH_4_^+^ but facilitating the diffusion of NH_3_ [23].

At present, majority of reports in the literature focused predominantly on *rhgp*/Rhgp in the gills of teleosts and there is no information on *rhgp*/Rhgp in lungfishes. Therefore, this study was undertaken to sequence the cDNA coding region of *rhgp* isoforms from the gills of *P*. *annectens*. It was hoped that the deduced Rhgp amino acid sequences would shed light on the phylogenetic relationships between *P*. *annectens* and other animals. Efforts were made to determine, using quantitative real-time polymerase chain reaction (qPCR), the mRNA expression levels of various *rhgp* in the gills of *P*. *annectens* after 6 days (the induction phase) or 6 months (the maintenance phase) of aestivation, or after 1 day or 3 days of arousal (the arousal phase) from 6 months of aestivation. Based on the deduced Rhgp sequences, custom-made anti-Rhgp antibodies were developed for the determination of the protein abundance of Rhgp in the gills of *P*. *annectens* through Western blotting. It was hypothesized that the branchial mRNA and protein expression of *rhgp*/Rhgp would be down-regulated to suppress ammonia excretion during the induction phase, and also to shut down branchial functions and thwart ammonia excretion during the maintenance phase. Furthermore, it was hypothesized that the branchial mRNA and protein expression of *rhgp*/Rhgp would be restored in order to regain the ability of ammonia excretion through the gills upon arousal from aestivation.

### Note on abbreviations

Two different types of abbreviations were adopted in this report because there are differences between the standard abbreviations of genes/proteins of fishes (http://zfin.org/cgi-bin/webdriver?MIval=aa-ZDB_home.apg) and those of frogs and human/non-human primates (http://www.genenames.org). For fishes, gene symbols are italicized, all in lower case, and protein designations are the same as the gene symbol, but not italicized with the first letter in upper case.

## Materials and methods

### Animals

This study was performed on the African lungfish *P*. *annectens* (Order Lepidosireniformes; Family Protopteridae). Specimens of *P*. *annectens* (80–150 g body mass) were collected from Central Africa and imported through a local fish farm in Singapore (Qian Hu Fish Farm Trading Co, Singapore). They were maintained in plastic aquaria filled with dechlorinated tap water at 25°C in the laboratory. Water was changed daily. No attempt was made to separate the sexes. Fish were acclimated to laboratory conditions for at least two weeks. During the acclimatization period, fish were fed with frozen fish meat.

## Ethics statement

The Institutional Animal Care and Use Committee (IACUC) of the National University of Singapore has specifically approved this study and the procedures used in this study (IACUC 035/09). All efforts were made to minimize the suffering of the lungfish.

### Experimental conditions and collection of samples

Food was withdrawn 96 h before experiments (day 0) for both control and aestivating lungfish, which gave sufficient time for the gut to be emptied of all food and waste. Lungfish (*N* = 5) kept in fresh water served as controls and were killed with an overdose of neutralized 0.05% MS222 for tissue sampling. Following the procedure of Chew et al. [4], some lungfish were induced to aestivate at 27–29°C and 85–90% humidity individually in plastic tanks (L29 cm x W19 cm x H17.5 cm) containing 15 ml dechlorinated tap water (made 0.3‰ with seawater). It took approximately 6 days for the lungfish to be encased in a brown dried mucus cocoon and these 6 days were counted as part of the aestivation period. The lungfish were allowed to aestivate for 6 months. In order to maintain a high humidity (>90%) within the tank, 1–2 ml of water was sprayed onto the side of the tank daily. After 6 months of aestivation, some lungfish were aroused by adding 200 ml of water into the tank and breaking up the cocoon manually. After a few minutes, the lungfish would swim sluggishly in the water; another 800 ml of water was added to cover the lungfish. Some lungfish were pithed after a blow to the head for tissue sampling after 6 days (the induction phase), or after 6 months (the maintenance phase) of aestivation (*N* = 5 for each group). Some lungfish were killed with an overdose of neutralized 0.05% MS222 after 1 or 3 days of arousal from 6 months of aestivation (the arousal phase) without food (*N* = 5 for each group). The eye, brain, gills, heart, liver, spleen, pancreas, gut, kidney, lung, muscle and skin were quickly excised and freeze-clamped with aluminum tongs pre-cooled in liquid nitrogen.

### Total RNA extraction and cDNA synthesis

Total RNA was extracted from all the samples of *P*. *annectens* using Tri Reagent (Sigma-Aldrich Co., St. Louis, MO, USA), and purified using the Qiagen RNeasy Mini Kit (Qiagen GmbH, Hilden, Germany). RNA was quantified spectrophotometrically using a BioSpec-nano (Shimadzu, Tokyo, Japan). The integrity of RNA was assessed electrophoretically and gauged by the ratio of 28S/18S rRNA. For polymerase chain reaction (PCR), first strand cDNA synthesis was performed using 4 μg of total RNA, oligo(dT)_18_ primer and the RevertAid first strand cDNA synthesis kit (Thermo Fisher Scientific, Waltham, MA, USA). For rapid amplification of cDNA ends (RACE), 5’-RACE-Ready cDNA and 3’-RACE-Ready cDNA were synthesized from 1 μg of total RNA using SMARTer RACE cDNA Amplification kit (Clontech Laboratories, Mountain View, CA, USA).

For qPCR, Qiagen RNeasy Plus Mini Kit (Qiagen GmbH), which contains gDNA Eliminator spin column (Qiagen GmbH) to remove genomic DNA, was used to purify the RNA from the gills of *P*. *annectens*. First strand cDNA synthesis was performed using 4 μg of total RNA, random hexamer primers and RevertAid first strand cDNA synthesis kit (Thermo Fisher Scientific).

### PCR and RACE

Partial sequences of *rhag*, *rhbg* and *rhcg* were obtained using gene-specific primers (S1 Table) designed from the highly conserved regions based on multiple alignments of the respective sequences from various fish species available in Genbank (http://www.ncbi.nlm.nih.gov/Genbank/). PCR and RACE was performed, following the procedure of Chng et al. [24], using the respective gene-specific PCR and RACE primers (S1 Table). PCR products were separated by gel electrophoresis. Bands of estimated molecular masses were excised and purified by FavorPrep Gel Purification Mini Kit (Favorgen Biotech Corp., Ping Tung, Taiwan). Sequencing was performed by a 3130XL Genetic Analyzer (Life Technologies Corporation, Carlsbad, California), using BigDye Terminator v3.1 Cycle Sequencing Kit (Life Technologies Corporation). Multiple sequencing was performed in both 3’ and 5’ directions to obtain the full coding sequence. Sequence assembly and analysis were performed using Bioedit v7.1.3 [25]. The complete coding cDNA sequences of *rhag*, *rhbg* and *rhcg* from *P*. *annectens* have been deposited to Genbank with accession numbers KX583645, KX583646 and KX583647, respectively.

In order to detect the mRNA expression levels of each gene in various tissues, PCR was also performed on the cDNAs of eye, brain, gills, heart, liver, spleen, pancreas, gut, kidney, lung, muscle, and skin of *P*. *annectens* following the procedure of Chng et al. [24].

### Deduced amino acid sequences and dendrogramic analyses

The deduced amino acid sequences of Rhag, Rhbg and Rhcg were translated from the corresponding nucleotide sequences using ExPASy Proteomic server (http://web.expasy.org/translate/). The deduced amino acid sequences were aligned and compared with selected Rhag/RhAG, Rhbg/RhBG and Rhcg/RhCG from various animal species using BioEdit. The transmembrane domains of Rhag, Rhbg and Rhcg of *P*. *annectens* were identified using MEMSAT3 and MEMSAT-SVM provided by PSIPRED protein structure prediction server (http://bioinf.cs.ucl.ac.uk/psipred/) [26]. Potential phosphorylation sites were identified using NetPhos 2.0, and potential *N*-glycosylation sites were identified using NetNGlyc 1.0.

The sequences of Rhag, Rhbg and Rhcg were aligned using ClustalX2 and dendrogramic analyses were performed using neighbor-joining method and 100 bootstrap replicates with Phylip [27]. The accession numbers of selected amino acid sequences of Rhag/RhAG, Rhbg/RhBG and Rhcg/RhCG (from GenBank) used in the dendrogramic analyses are indicated in S2, S3 and S4 Tables, respectively.

### qPCR

In this study, the method of relative quantification (2^-ΔΔCt^) was adopted, whereby the mRNA expression levels of target genes were compared with that of a reference gene within the same sample [28]. The 2^-ΔΔCt^ equation, including the assumptions and validation tests [28], were used to validate the reference gene and analyze the qPCR data. The reference gene adopted for 2^-ΔΔCt^ analysis was *sec62 homolog*, *preprotein translocation factor* (*sec62*). The mRNA expression of *rhag*, *rhbg* or *rhcg* were first normalized to the mRNA expression of *sec62* and then compared with the control lungfish and expressed as 2^-ΔΔCt^. Logarithmic transformation (log_2_) of the mean fold-change values were used for statistical analyses.

qPCR was performed in duplicates using a StepOnePlus Real-Time PCR System (Life Technologies Corporation), following the procedure of Chng et al. [24]. The mRNA expression levels of *rhag*, *rhbg*, *rhcg* and *sec62* were determined using gene-specific qPCR primers (S1 Table). The qPCR reactions contained 5 μl of KAPA SYBR FAST Master Mix (2X) ABI Prism (Kapa Biosystems, Woburn, MA, USA), 0.3 μmol l^-1^ of forward and reverse qPCR primers each and 1 ng of sample cDNA in a total volume of 10 μl. The PCR efficiencies for *rhag*, *rhbg*, *rhcg* and *sec62* were 101.5%, 95.3%, 93.3% and 97.1%, respectively.

### SDS-PAGE and Western blotting

A commercial firm (GenScript, Piscataway, NJ, USA) was engaged to raise a rabbit polyclonal antibody against aa 198‒211 (CYRSGLRNGHDNEGS) of Rhag, a rabbit polyclonal antibody against aa 30‒43 (FVRYDKETDPKEWH) of Rhbg and a rabbit polyclonal antibody against aa 29‒42 (IRYSDEADAARWPE) of Rhcg from the gills of *P*. *annectens*. Immunoreactive bands of Rhag, Rhbg and Rhcg were visualized at the expected molecular mass of 46.8 kDa, 50.9 kDa and 50.9 kDa, respectively.

Recombinant proteins of Rhag, Rhbg and Rhcg from *P*. *annectens* were synthesized from 1 μg of complete coding cDNA fragments of *rhag*, *rhbg* and *rhcg*, respectively, using 1-Step Human Coupled IVT Kit—DNA (Thermo Fisher Scientific) according to manufacturer’s instruction. The recombinant proteins were used to verify the specificity of the anti-Rhag, anti-Rhbg and anti-Rhcg antibodies. The complete coding cDNA fragments of *rhag*, *rhbg* and *rhcg* were generated by PCR (IVT) primers (S1 Table). The reaction mixtures were incubated at 30°C for 6 h and diluted with Laemmli buffer. The recombinant proteins were heated at 70°C for 15 min, and then kept at -80°C until analysis.

Following the procedure of Chng et al. [24], western blotting was performed on the recombinant proteins of Rhag, Rhbg and Rhcg, and the gill samples obtained from the control fish and fish that had undergone 6 days or 6 months of aestivation, or 1 or 3 days of arousal from 6 months of aestivation. The concentrations of the anti-Rhag, anti-Rhbg and anti-Rhcg antibodies used for Western blotting were 2 μg ml^−1^. The protein loads of the gill samples for gel separation were 100 μg for Rhag, 100 μg for Rhbg and 50 μg for Rhcg. Aestivation involves a complex interplay between up-regulation and down-regulation of diverse cellular activities [29], whereby many genes and/or proteins clusters are strategically up-regulated or down-regulated to meet the challenges associated with aestivation. At present, we were unable to identify an appropriate reference protein, the expression of which would be unaffected throughout the three phases of aestivation, for Western blotting. Hence, results were expressed as arbitrary densitometric unit per μg protein, i.e. with reference to the total protein abundance, which has been reported previously for several other proteins from *P*. *annectens* during aestivation [24,29–34].

### Statistical analyses

Results were presented as means ± standard errors of the mean (S.E.M.). Statistical analyses were performed using SPSS version 18 (SPSS Inc, Chicago, USA). Homogeneity of variance was checked using Levene’s Test. Differences between means were tested using one-way analysis of variance followed by multiple comparisons of means by either the Tukey or Dunnett T3 post-hoc test, depending on the homogeneity of variance of the data set. Differences with *P*<0.05 were reported as statistically significant.

## Results

### The nucleotide and the deduced amino acid sequences of *rhag*/Rhag, *rhbg*/Rhbg and *rhcg*/Rhcg

The complete coding cDNA sequences of *rhag*, *rhbg* and *rhcg* had been obtained from the gills of *P*. *annectens*. The coding sequence of *rhag* (accession: KX583645) consisted of 1311 bp, coding for 436 amino acids with an estimated molecular mass of 46.8 kDa, and that of *rhbg* (accession: KX583646) consisted of 1398 bp, coding for 465 amino acids with an estimated molecular mass of 50.9 kDa. For *rhcg*, the coding sequence (accession: KX583647) consisted of 1386 bp, coding for 461 amino acids with an estimated molecular mass of 50.9 kDa.

A hydropathy analysis demonstrated that the deduced amino acid sequences of Rhag, Rhbg and Rhcg of *P*. *annectens* comprised 12 transmembrane regions. Efforts were made to compare the three Rhgp of *P*. *annectens* with *Escherichia coli* ammonia transporter (AmtB), as its high resolution crystal structure had been resolved and based on which theories of NH_3_/NH_4_^+^ by Rhgp had been formulated [35,36].

**Fig 1:**
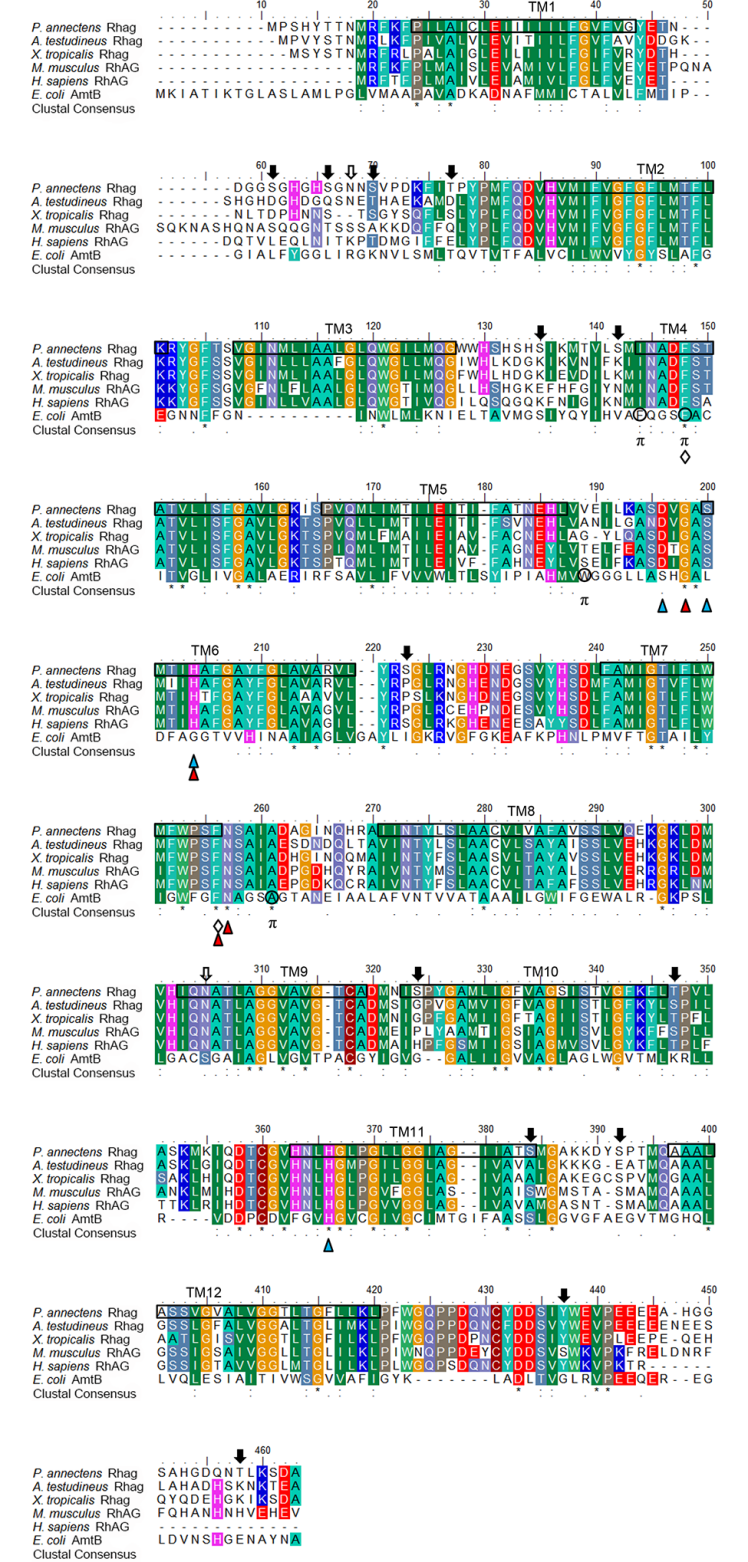
Molecular characterization of Rhesus blood group-associated glycoprotein (Rhag) from the gills of *Protopterus annectens*. A multiple amino acid alignment of Rhag from *Protopterus annectens* with *Anabas testudineus* Rhag (AIC81181.1), *Xenopus (Silurana) tropicalis* Rhag (XP_002933645.2), *Mus musculus* RhAG (AAI01942.1), *Homo sapiens* RhAG (NP_000315.2) and *Escherichia coli* ammonia transporter (AmtB; NP_414985.1). Identical amino acid residues are indicated by asterisks, strongly similar amino acids are indicated by colons and weakly similar amino acids are indicated by periods. Residues involved in NH_4_^+^ binding and deprotonation of NH_4_^+^ for NH_3_ conduction are indicated by red and blue triangles, respectively. The phenylalanine gate is indicated by open diamonds. The π cation binding sites of *E*. *coli* AmtB are indicated by “π”. Potential *N*-glycosylation and phosphorylation sites are indicated by open and shaded arrows, respectively. The predicted transmembrane domains (TM1‒12) of Rhag of *P*. *annectens* are indicated by open boxes and were predicted using MEMSATS and MEMSAT-SVA provided by PSIPRED protein structure prediction server.

An alignment of Rhag from *P*. *annectens* with *E*. *coli* AmtB and Rhag/RhAG from human, mouse, frog and fish revealed highly conserved residues involved in gating of the channel (F148 and F256), NH_4_^+^ binding (G198, H204, F256 and N257) and deprotonation of NH_4_^+^ for NH_3_ conduction (D196, S200, H204 and H366, Fig 1). Rhag of *P*. *annectens* lacked the potential π cation binding sites of *E*. *coli* AmtB (Fig 1). There were 13 potential phosphorylation sites and 2 *N*-glycosylation sites in Rhag of *P*. *annectens* (Fig 1). A comparison of Rhag of *P*. *annectens* with Rhag/RhAG of other animal species indicated that it had the highest sequence similarity with amphibian Rhag (70.1–73.1%), followed by those of actinopterygians (69.1–71.7%) and mammals (59.6–64.9%; S5 Table).

**Fig 2:**
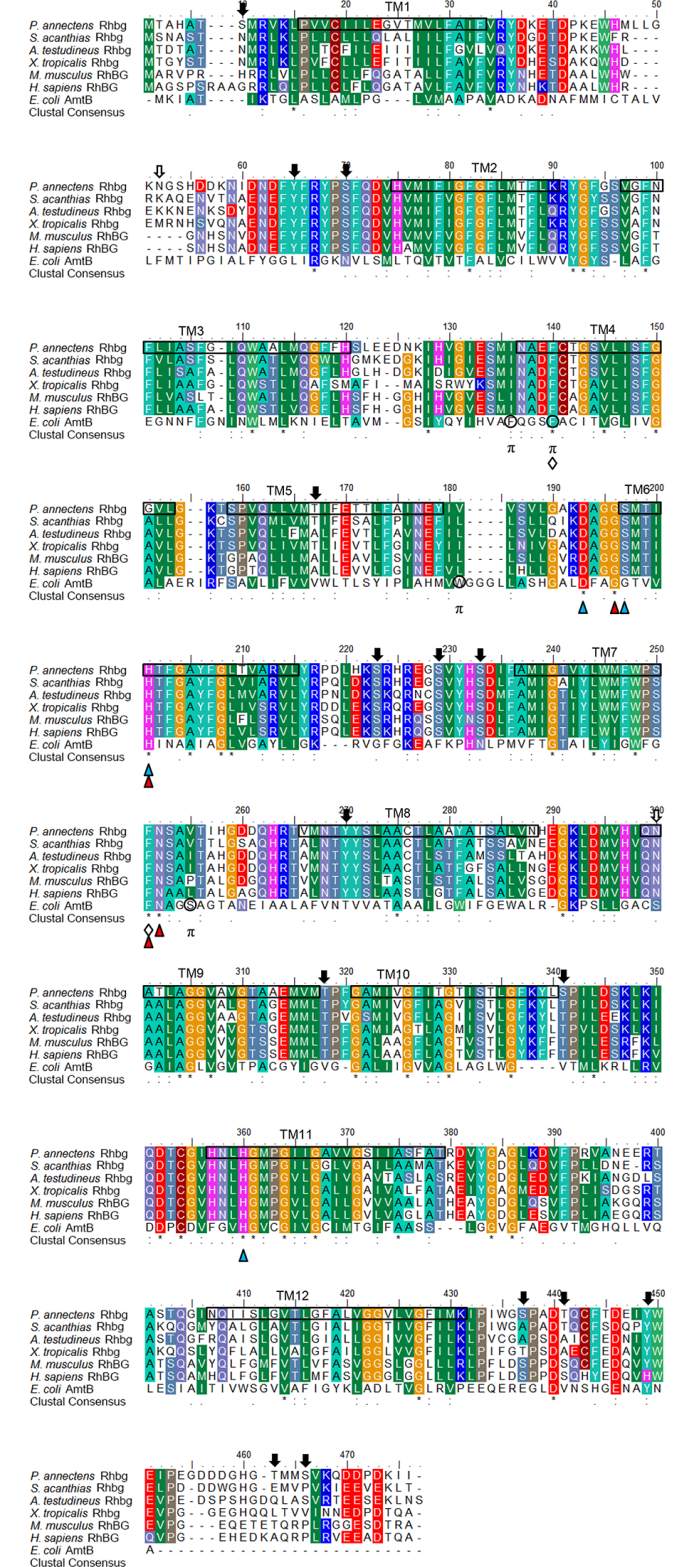
Molecular characterization of Rhesus family B glycoprotein (Rhbg) from the gills of *Protopterus annectens*. A multiple amino acid alignment of Rhbg from *Protopterus annectens* with *Squalus acanthias* Rhbg (AJF44128.1), *Anabas testudineus* Rhbg (AIC81182.1), *Xenopus (Silurana) tropicalis* Rhbg (AAU89493.1), *Mus musculus* RhBG (AAF19371.1), *Homo sapiens* RhBGA (NP_000315.2) and *Escherichia coli* ammonia transporter (AmtB; NP_414985.1). Identical amino acid residues are indicated by asterisks, strongly similar amino acids are indicated by colons and weakly similar amino acids are indicated by periods. Residues involved in NH_4_^+^ binding and deprotonation of NH_4_^+^ for NH_3_ conduction are indicated by red and blue triangles, respectively. The phenylalanine gate is indicated by open diamonds. The π cation binding sites of *E*. *coli* AmtB are indicated by “π”. Potential *N*-glycosylation and phosphorylation sites are indicated by open and shaded arrows, respectively. The predicted transmembrane domains (TM1‒12) of Rhbg of *P*. *annectens* are indicated by open boxes and were predicted using MEMSATS and MEMSAT-SVA provided by PSIPRED protein structure prediction server.

An alignment of Rhbg of *P*. *annectens* with *E*. *coli* AmtB and Rhbg/RhBG from human, mouse, frog and two fishes (*Squalus acanthias* and *Anabas testudineus*) showed that the residues involved in gating of the channel (F140 and F251), NH_4_^+^ binding (G196, H201, F251 and N252) and deprotonation of NH_4_^+^ for NH_3_ conduction (D193, S197, H201 and H360) were highly conserved (Fig 2). Rhbg of *P*. *annectens* lacked the potential π cation binding sites of *E*. *coli* AmtB (Fig 2). There were 15 potential phosphorylation sites and 2 *N*-glycosylation sites present in Rhbg of *P*. *annectens* (Fig 2). A comparison of Rhbg of *P*. *annectens* with Rhbg/RhBG of other animal species indicated that it had the highest sequence similarity with chondrichthyes Rhbg (64.9–69.2%), followed by those of actinopterygians (63.5–69.0%), amphibians (66.4–68.2%) and mammals (49.4–58.1%; S6 Table).

**Fig 3:**
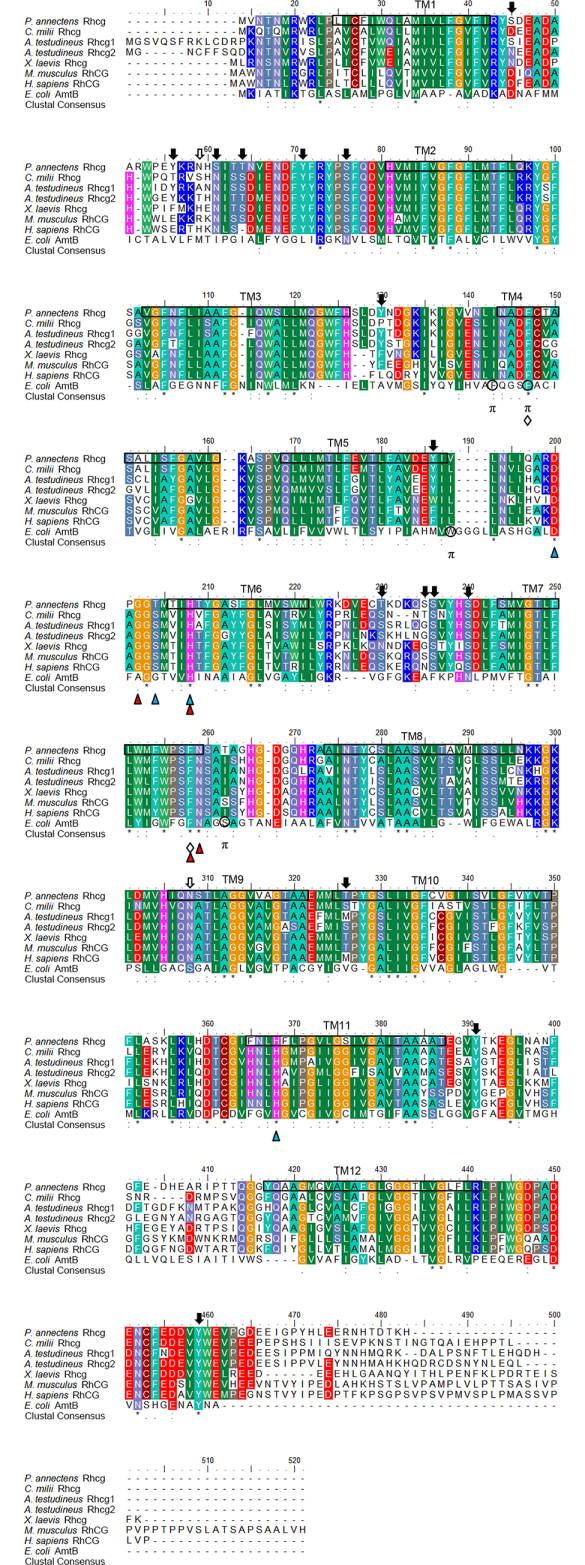
Molecular characterization of Rhesus family C glycoprotein (Rhcg) from the gills of *Protopterus annectens*. A multiple amino acid alignment of Rhcg from *Protopterus annectens* with *Callorhinchus milii* Rhcg (AFO96383.1), *Anabas testudineus* Rhcg1 (AIC81183.1) and Rhcg2 (AIC81184.1), *Xenopus laevis* Rhcg (NP_001088553.1), *Mus musculus* RhCG (AAF19373.1), *Homo sapiens* RhCG (AAF19372.1) and *Escherichia coli* ammonia transporter (AmtB; NP_414985.1). Identical amino acid residues are indicated by asterisks, strongly similar amino acids are indicated by colons and weakly similar amino acids are indicated by periods. Residues involved in NH_4_^+^ binding and deprotonation of NH_4_^+^ for NH_3_ conduction are indicated by red and blue triangles, respectively. The phenylalanine gate is indicated by open diamonds. The π cation binding sites of *E*. *coli* AmtB are indicated by “π”. Potential *N*-glycosylation and phosphorylation sites are indicated by open and shaded arrows, respectively. The predicted transmembrane domains (TM1‒12) of Rhcg of *P*. *annectens* are indicated by open boxes and were predicted using MEMSATS and MEMSAT-SVA provided by PSIPRED protein structure prediction server.

An alignment of Rhcg of *P*. *annectens* with *E*. *coli* AmtB and Rhcg/RhCG from human, mouse, frog and two fishes (*Callorhinchus milii* and *A*. *testudineus*) revealed that the residues involved in gating of the channel (F147 and F258) and NH_4_^+^ binding (G202, H208, F258 and N259) were conserved (Fig 3). The residues involved in the deprotonation of NH_4_^+^ for NH_3_ conduction (D200, H208 and H368) are highly conserved, except for a replacement of T204 in *P*. *annectens* Rhcg with S204 in human RhCG (Fig 3). Rhcg of *P*. *annectens* lacked the potential π cation binding sites of *E*. *coli* AmtB (Fig 3). There were 15 potential phosphorylation sites and 2 *N*-glycosylation sites in Rhcg of *P*. *annectens* (Fig 3). A comparison of Rhcg of *P*. *annectens* with Rhcg/RhCG of other animal species indicated that it had the highest sequence similarity with actinopterygian Rhcg (60.2–65.7%), followed by those of *C*. *milii* (64.2%), amphibians (63.4–64.1%) and mammals (55.4–61.5%; S7 Table).

### The dendrogramic analyses of Rhag, Rhbg and Rhcg

**Fig 4:**
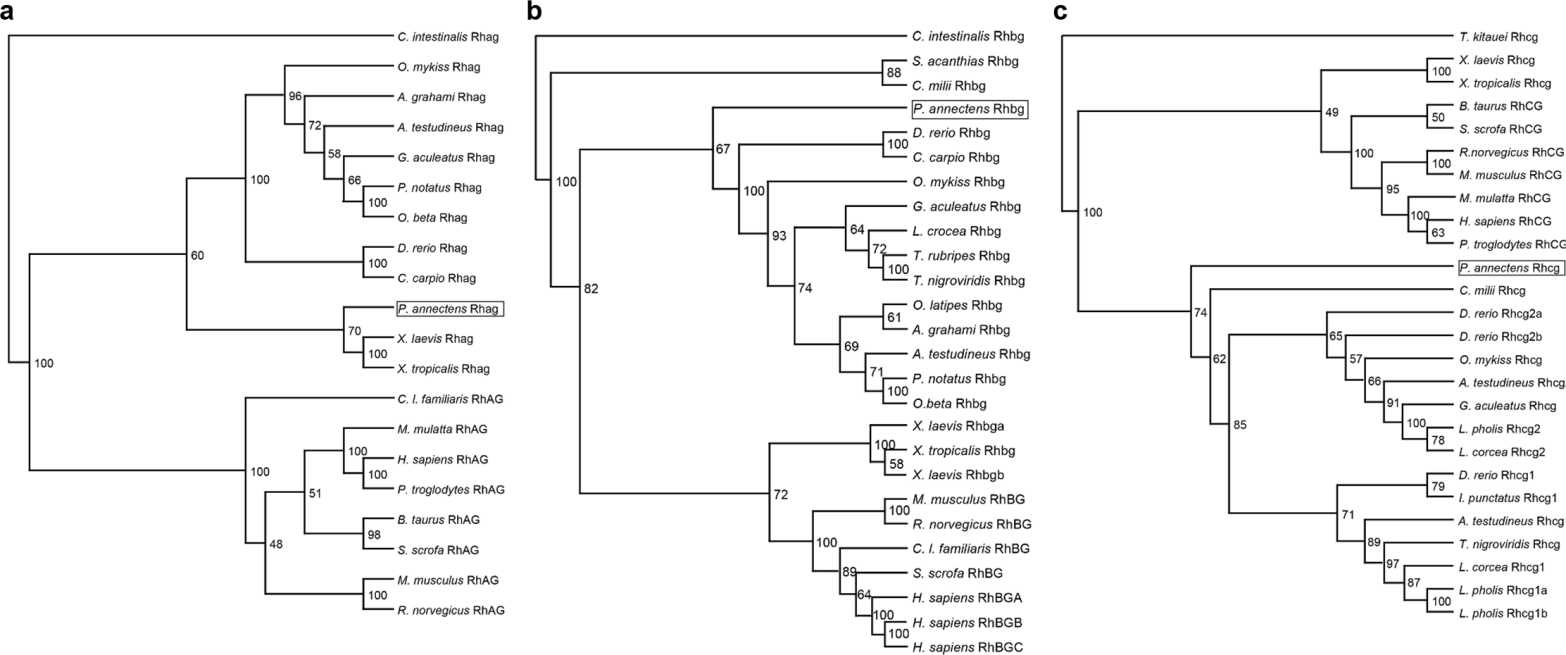
**Dendrograms of (a) Rhesus blood group-associated glycoprotein (Rhag/RhAG), (b) Rhesus family B glycoprotein (Rhbg/RhBG) and (c) Rhesus family C glycoprotein (Rhcg/RhCG) including those of *Protopterus annectens*.** Numbers presented at each branch point represent bootstrap values from 100 replicates. *Ciona intestinalis* Rhag and Rhbg and *Thelohanellus kitauei* Rhcg are used as outgroups for their respective dendrogram.

Rhag of *P*. *annectens* was grouped together with amphibian Rhag, separated from the mammal and actinopterygian clades (Fig 4A). By contrast, Rhbg and Rhcg of *P*. *annectens* were grouped together with actinopterygian Rhbg (Fig 4B) and Rhcg (Fig 4C), respectively, separated from the tetrapods.

### The mRNA expression levels of *rhag*, *rhbg* and *rhcg* in various tissues/organs of *P*. *annectens*

**Fig 5:**
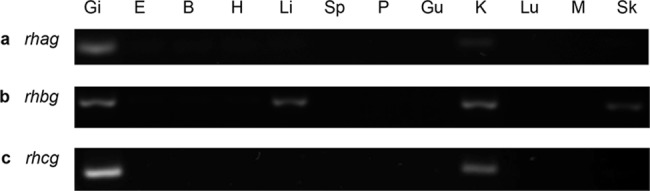
**mRNA expression of (a) *rhesus blood group-associated glycoprotein* (*rhag*), (b) *rhesus family B glycoprotein* (*rhbg*) and (c) *rhesus family C glycoprotein* (*rhcg*) in various tissues/organs of *Protopterus annectens***. The mRNA expression of *rhag*, *rhbg* and *rhcg* were examined in the gills (Gi), eyes (E), brain (B), heart (H), liver (Li), spleen (Sp), pancreas (P), gut (Gu), kidney (K), Lung (Lu), muscle (M) and skin (Sk) of *P*. *annectens* kept in fresh water.

For *P*. *annectens* kept in fresh water, *rhag* was expressed in the gills (Fig 5A), *rhbg* was expressed in the gills, liver and kidney (Fig 5B), and *rhcg* was expressed in the gills and kidney (Fig 5C).

### Effects of aestivation on the mRNA expression levels of *rhag*, *rhbg* and *rhcg* in the gills of *P*. *annectens*

**Fig 6:**
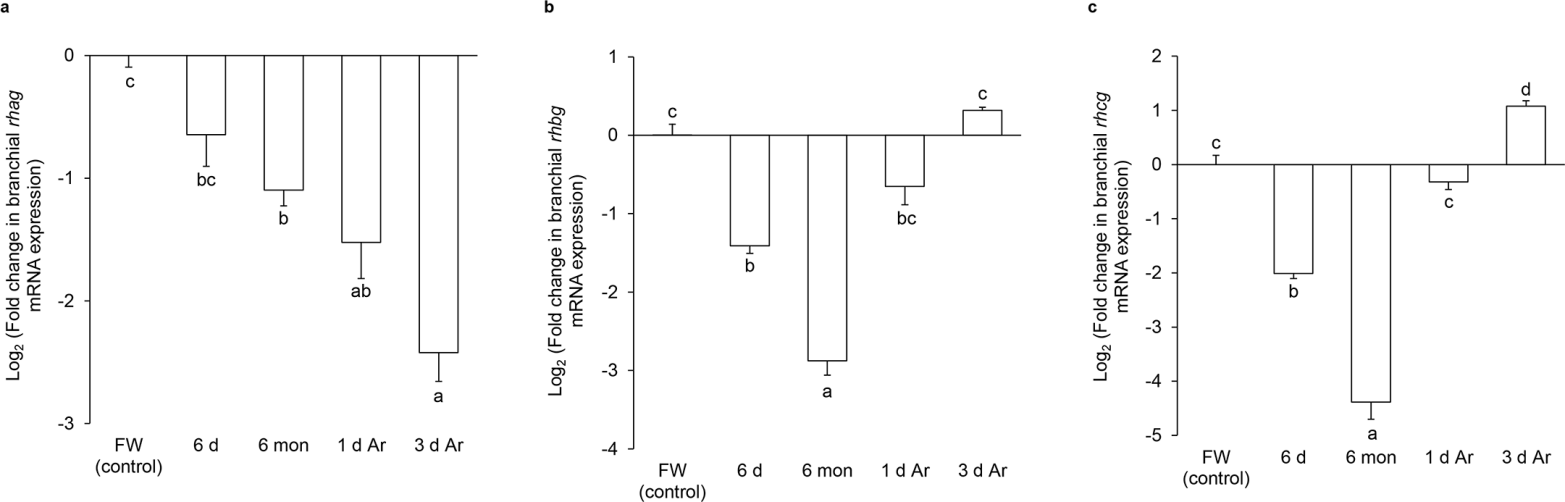
mRNA expression levels of *rhesus glycoproteins* in the gills of *Protopterus annectens*. Relative mRNA expression (determined using qPCR and calculated based on 2^-ΔΔCT^) of (a) *rhesus blood group-associated glycoprotein* (*rhag*), (b) *rhesus family B glycoprotein* (*rhbg*) and (c) *rhesus family C glycoprotein* (*rhcg*) in the gills of *Protopterus annectens* kept in fresh water on day 0 (FW; control), after 6 days (d; the induction phase) or 6 months (mon; the maintenance phase) of aestivation, or after 1 or 3 d of arousal (Ar; the arousal phase) from 6 mon of aestivation. Results represent means ± S.E.M (*N* = 5). Means not sharing the same letter are significantly different (*P* < 0.05).

In order to determine the mRNA expression levels of *rhag*, *rhbg* and *rhcg* in the gills of *P*. *annectens* during the three phases of aestivation, qPCR based on the ΔΔCt method was performed. The mRNA expression level of *rhag* remained unchanged in the gills of *P*. *annectens* after 6 days of aestivation as compared with the control. However, it decreased significantly after 6 months (by 53%) of aestivation, or after 1 day (by 65%) or 3 days (by 81%) of arousal from 6 months of aestivation (Fig 6A).

The branchial mRNA expression level of *rhbg* decreased significantly after 6 days (by 62%) or 6 months (by 86%) of aestivation, but returned to the control level after 1 day of arousal (Fig 6B).

As for *rhcg*, its mRNA expression level decreased significantly in the gills of *P*. *annectens* after 6 days (by 75%) or 6 months (by 95%) of aestivation as compared to control fish (Fig 6C), but it returned to the control level after 1 day of arousal and became significantly higher (2.1-fold) than the control after 3 days of arousal from 6 months of aestivation (Fig 6C).

### Effects of aestivation on the protein abundance of Rhag, Rhbg and Rhcg in the gills of *P*. *annectens*

**Fig 7:**
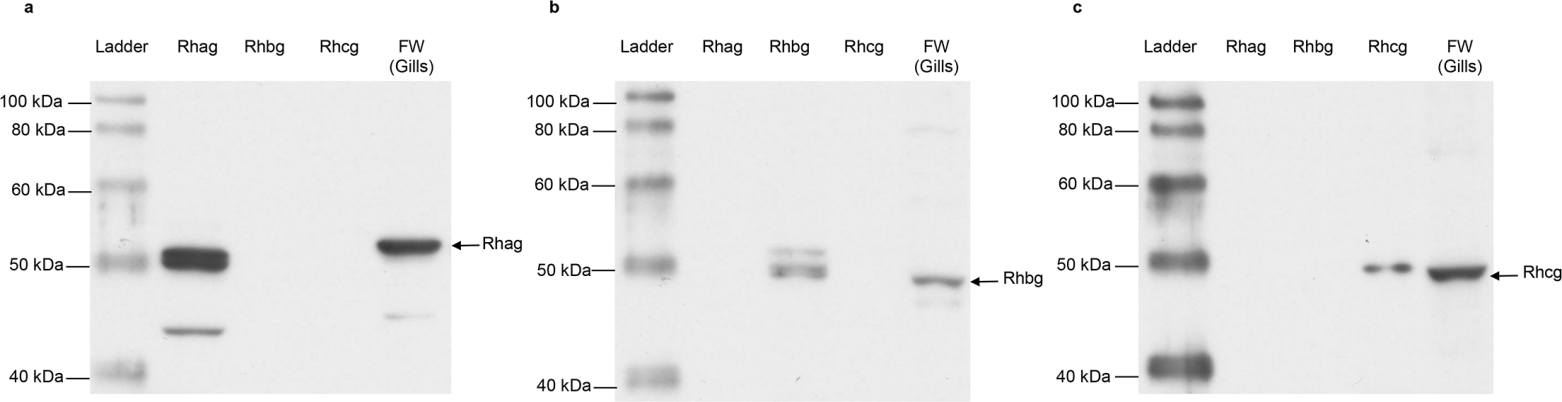
Validation of anti-Rhesus glycoproteins antibodies from *Protopterus annectens*. Immunoblots incubated with (a) anti-Rhesus blood group-associated glycoprotein (Rhag), (b) anti-Rhesus family B glycoprotein (Rhbg) and (c) anti-Rhesus family C glycoprotein (Rhcg) antibodies. Recombinant proteins of Rhag, Rhbg, Rhcg and the protein from the gills of *Protopterus annectens* kept in fresh water on day 0 (FW) were loaded for gel separation.

**Fig 8:**
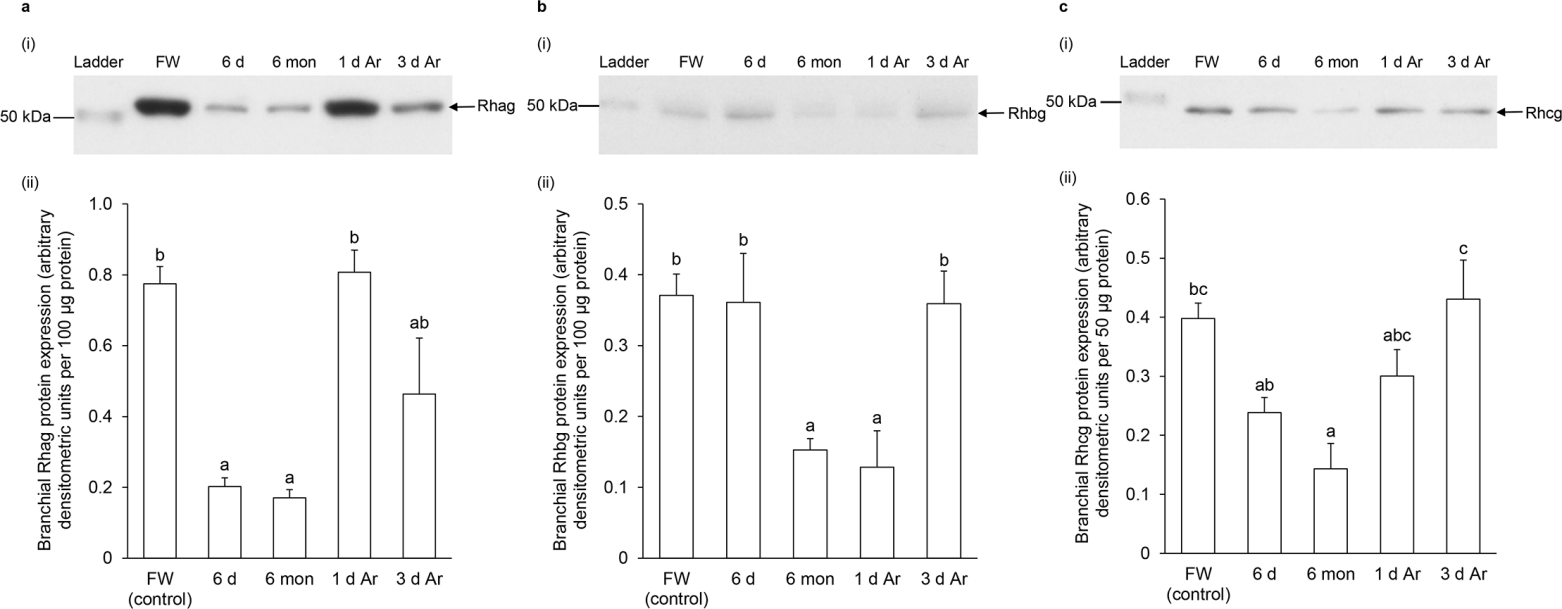
Protein abundance of Rhesus glycoproteins in the gills of *Protopterus annectens*. Protein abundance of (a) Rhesus blood group-associated glycoprotein (Rhag), (b) Rhesus family B glycoprotein (Rhbg) and (c) Rhesus family C glycoprotein (Rhcg) in the gills of *Protopterus annectens* kept in fresh water on day 0 (FW; control), or after 6 days (d; the induction phase) or 6 months (mon; the maintenance phase) of aestivation, or after 1 or 3 d of arousal (Ar; the arousal phase) from 6 mon of aestivation. (i) Examples of immunoblot of Rhag, Rhbg or Rhcg. (ii) The protein abundance of Rhag, Rhbg or Rhcg expressed as arbitrary densitometric units per 100 μg, 100 μg or 50 μg protein, respectively. Results represent mean ± S.E.M. (*N* = 4). Means not sharing the same letter are significantly different (*P*<0.05).

Western blotting using the custom-made anti-Rhag antibody revealed a band at ~50 kDa which is close to the estimated molecular mass of the deduced Rhag sequence of *P*. *annectens* (Fig 7A). The anti-Rhag antibody recognized only Rhag recombinant protein and showed no cross-reactivity with Rhbg and Rhcg recombinant proteins (Fig 7A), thus validating the antibody specificity to Rhag. The protein abundance of Rhag decreased significantly in the gills of *P*. *annectens* after 6 days (74%) or 6 months (by 78%) of aestivation, but returned to the control level after 1 day or 3 days of arousal from 6 months of aestivation (Fig 8A).

Western blotting using the custom-made anti-Rhbg antibody revealed a band at ~48 kDa which is close to the estimated molecular mass of the deduced Rhbg sequence of *P*. *annectens* (Fig 7B). The anti-Rhbg antibody recognized only Rhbg recombinant protein and showed no cross-reactivity with Rhag and Rhcg recombinant proteins (Fig 7B), thus validating the antibody specificity to Rhbg. The protein abundance of Rhbg remained unchanged in the gills of *P*. *annectens* after 6 days of aestivation, but decreased significantly after 6 months (by 59%) of aestivation, or after 1 day (by 65%) of arousal from 6 months of aestivation (Fig 8B). The branchial protein abundance of Rhbg returned to the control level on day 3 of arousal (Fig 8B).

Western blotting using the custom-made anti-Rhcg antibody revealed a band at ~48 kDa which is close to the estimated molecular mass of the deduced Rhcg sequence of *P*. *annectens* (Fig 7C). The anti-Rhcg antibody recognized only Rhcg recombinant protein and showed no cross-reactivity with Rhag and Rhbg recombinant proteins (Fig 7C), thus validating the antibody specificity to Rhcg. The protein abundance of Rhcg remained unchanged in the gills of *P*. *annectens* after 6 days of aestivation (Fig 8C). However, it decreased significantly (by 64%) after 6 months of aestivation as compared to control fish, and returned to the control level after 1 day or 3 days of arousal (Fig 8C).

## Discussion

### Molecular characterization of Rhgp of *P*. *annectens*

Rhag, Rhbg and Rhcg of *P*. *annectens* comprise 12 transmembrane domains, which are in agreement with the typical topology of fish Rhgp and mammalian RhGP [37–39]. Four structural motifs involved in (1) the gating of the channel, (2) deprotonation of NH_4_^+^ and NH_3_ conduction, (3) NH_4_^+^ binding and (4) π cation binding have been identified in *E*. *coli* AmtB and *H*. *sapiens* RhCG [35,40,41]. In AmtB of *E*. *coli*, F107 and F215 (corresponding to F130 and F235 in human RhCG) are involved in the gating of the channel which supply π-cation interaction for NH_4_^+^ [40]. Furthermore, transient movement of side chains of F130 and F235 in human RhCG is necessary for ammonia conductance [35]. For the three Rhgp from *P*. *annectens*, both phenylalanine residues (F148 and F256 in Rhag, F140 and F251 in Rhbg, and F147 and F258 in Rhcg) were conserved. In human RhCG, D177, S181, H185 and H344 are involved in the deprotonation of NH_4_^+^ for NH_3_ conduction [40,41]. These residues are conserved in Rhag and Rhbg of *P*. *annectens*, but for Rhcg of *P*. *annectens*, S181 in human RhCG is replaced with threonine. Baday et al. [41] proposed that S181 was not directly involved in the proton transfer but it played a structural role by maintaining D177 in position, allowing for the formation of a stable water chain between D177 and H185. Therefore, the change of S181 in human RhCG to threonine in Rhcg of *P*. *annectens* should not affect the deprotonation of NH_4_^+^ for NH_3_ conduction. Furthermore, based on molecular dynamics simulation, G179, H185, F235 and N236 in human RhCG are involved in NH_4_^+^ binding [41], and these four residues are conserved in the three Rhgp of *P*. *annectens*. F103, F107 and W148 are the π cation binding sites in *E*. *coli* AmtB. F107 in *E*. *coli* AmtB is conserved in the three Rhgp of *P*. *annectens*, but F103 and W148 in *E*. *coli* AmtB are replaced with isoleucine and valine in all the Rhgp of *P*. *annectens*, respectively. Notably, *E*. *coli* AmtB-W148L mutant increases the flux of NH_4_^+^ significantly with dependency on the electrochemical potential gradient across the membrane as compared to wild type AmtB [42]. If indeed Rhgp of *P*. *annectens* transport NH_4_^+^, the replacement of W148 in *E*. *coli* AmtB with smaller non-polar amino acids might potentially increase the flux of NH_4_^+^. Taken together, the four structural motifs are conserved in Rhgp of *P*. *annectens*, with minor amino acid substitutions at selected positions. The results indicate that Rhgp of *P*. *annectens* can deprotonate NH_4_^+^ and mediate the transport of NH_3_ through a hydrophobic pore. However, based on molecular characterization alone, a definite conclusion on the function of the Rhgp cannot be drawn [43,44]. Therefore, experimental testing remains essential to examine the function of the Rhgp of *P*. *annectens* and efforts should be made in the future to elucidate whether Rhgp of *P*. *annectens* could transport NH_3_ and/or NH_4_^+^.

### Dendrogramic analyses of Rhgp

Dendrogramic analyses indicated that Rhag was grouped closer to fish, while Rhbg and Rhcg were grouped closer to tetrapods. When taken together with the phylogenetic information based on the deduced amino acid sequences of other genes of *P*. *annectens*, our results are in agreement with the notion that lungfish is the intermediary form between fish and tetrapods. Since lungfishes are the closest living sister group of land vertebrates, they would logically possess some genes/proteins that are closer to those of other fishes (Cps III, [11]; Ass, [12]) and others that have greater similarity to those of tetrapods (Asl, [12]; L-gulono-γ-lactone oxidase, [30]; Na^+^/K^+^-ATPase α-subunit isoforms, [31]; Coagulation factor II and Fibrinogen gamma chain [32]; Betaine-homocysteine *S*-methyltransferase 1, [33]; Myostatin [34]; Aquaporin 1 and 3 [24]; Urea transporter isoforms [29]).

Huang and Peng [38] reported that Rh homologs cluster in subgroups and suggested that natural selection had acted differently on these subgroups. As a whole, Rhgp/RhGP are highly conserved, but its subgroups vary in divergence rate and sites which could be related to functional specification to species adaptation [45]. In invertebrates, there are negative selection on Rh proteins and a similar degree of sequence identity to the individual vertebrate subgroups, RhBG > RhCG > RhAG > Rh30 [45]. While there is negative selection on epithelial Rh homologs (Rhbg and Rhcg) in vertebrates, positive selection occurs to erythroid Rh proteins (Rh30 and Rhag) [46]. Therefore, erythroid Rh proteins diverge more rapidly compared to epithelial Rh proteins [46], accounting why Rhag of *P*. *annectens* is grouped closer to the tetrapods, while Rhbg and Rhcg of *P*. *annectens* are grouped closer to the fishes.

### Transcriptional and translational regulation of branchial *rhgp*/Rhgp expression during aestivation

After 6 days of aestivation, there was a significant decrease in the protein abundance of Rhag, but not the transcript level of *rhag*, in the gills of *P*. *annectens*. It is possible that there was a decrease in the translation of Rhag or an increase in its removal. Either way, it is logical to postulate that Rhag was regulated mainly at the protein level in the gills of *P*. *annectens*. This postulation is supported by the fact that, after 1 day or 3 days of arousal, the transcript level of *rhag* remained significantly lower than the control, but the protein abundance of Rhag had returned to the control level. By contrast, the regulation of Rhbg and Rhcg expression appears to involve both transcriptional and translational processes. In both cases, changes in protein abundance (translation) were preceded by corresponding changes in transcript levels (transcription). For instance, significant down-regulation in the mRNA expression levels of *rhbg* and *rhcg* were detected in the gills of *P*. *annectens* during the induction phase, but a significant decrease in the protein abundance of Rhbg and Rhcg occurred only during the maintenance phase of aestivation.

### Changes in branchial *rhgp*/Rhgp expression during the induction phase

In humans, *RhAG/*RhAG is expressed in erythrocytes and erythropoeitic tissues [22,39]. RhAG is involved in mediating ammonia transport so that erythrocytes are able to transport ammonia in the blood from its site of production to the site of excretion [47]. Since the gills of *P*. *annectens* were not perfused before sampling in this study, the gill samples naturally contained erythrocytes. The ammonia produced by the liver (and other organs) may be carried by the erythrocytes (and plasma) to the gills where ammonia is released through Rhag and transferred to the branchial epithelial cells for excretion. Therefore, the decrease in the protein abundance of Rhag in *P*. *annectens* during the induction phase might indicate a decrease in the release of ammonia from the erythrocytes to the plasma. It is possible that these erythrocytes carry a sub-normal level of ammonia due to a reduction in ammonia produced in the liver of *P*. *annectens*. It has been established that aestivation involves structural and functional modifications in certain organs during the induction phase [48–50]. These modifications involve the recycling of nitrogen between protein degradation and protein synthesis as the aestivating lungfish is undergoing fasting, which would lead to a reduction in the production of ammonia through amino acid catabolism [6,51]. Alternatively, Rhag has been localized to both the apical and basolateral membranes of the pillar cells in *Takifugu rubripes* [52], membranes that surrounds the lamellar blood spaces in the gills of *D*. *rerio* [53], and in the pillar cells, the lamellar epithelial cells and the outer edge of the interlamellar cell mass enriched with Na^+^/K^+^-ATPase in *Carassius auratus* [54]. If indeed Rhag is expressed in the gills of *P*. *annectens*, a decrease in the protein abundance of Rhag during the induction phase might lead to a reduction in ammonia excretion through the gills. Despite decreases in the mRNA expression levels of *rhbg* and *rhcg*, the protein abundance of Rhbg and Rhcg remained unchanged during the induction phase. These can be regarded as the necessary preparation to shut down gill functions and to stop ammonia excretion as the fish entered into the maintenance phase.

### Changes in branchial *rhgp*/Rhgp expression during the maintenance phase

The gills of *P*. *annectens* apparently cease to function during the maintenance phase, as they are covered with a thick layer of dried mucus with a reduction in the surface area of the branchial epithelium [16]. Indeed, ammonia excretion, which occurs mainly through the gills, stops completely in *P*. *annectens* during the maintenance phase. Should this be regarded as a passive response to a lack of water to flush the branchial surface and/or a physiological adaptation which involves molecular changes in the gills? Here we demonstrate that arrest of physiological functions (e.g. ammonia excretion) occurs in association with changes in the expression of the related transporters/channels (e.g. *rhgp*/Rhgp). The decreased branchial transcript levels and protein abundance of *rhag*/Rhag, *rhbg*/Rhbg and *rhcg*/Rhcg during the maintenance phase indicate that the transcription and translation of these *rhgp*/Rhgp were down-regulated. This can be interpreted as part of an overall mechanism to shut down branchial functions. As it is essential to conserve metabolic fuels during long periods of aestivation without food intake, and as transcription and translation are energy-dependent processes [55], the suppression of transcription and translation of *rhgp*/Rhgp would certainly reduce the expenditure of metabolic energy. Therefore, these results support the notion that aestivation involves structural and functional modifications in certain organs [48–50], and the complex interplay between up-regulation and down-regulation of diverse cellular activities during different phases of aestivation [1,3].

Based on models of Rhgp in many fishes [22,23,39], Rhbg and Rhcg are localized to the basolateral and apical membrane of branchial epithelial cells, respectively. Nakhoul and Hamm [18] proposed that Rhbg and Rhcg were likely to work in tandem and function as NH_3_ channel and/or NH_4_^+^-specific transporter, facilitating transepithelial ammonia transport. Therefore, the decrease in the protein abundance of Rhbg and Rhcg in the gills of *P*. *annectens* during the maintenance phase would lead to a decrease in the transepithelial movement of ammonia. Furthermore, the decrease in the protein abundance of Rhag might imply a reduction in the transfer of ammonia from the erythrocytes to the branchial epithelium. Hence, efforts should be made in the future to elucidate the cellular and/or subcellular localization of Rhag, Rhbg and Rhcg in the gills of *P*. *annectens* in order to substantiate their possible functions in transepithelial ammonia transport.

### Changes in *rhgp*/Rhgp expression during the arousal phase

During the arousal phase, it is essential for the lungfish to regain the ability to excrete ammonia. Indeed, the protein abundance of Rhag, Rhbg and Rhcg recovered to the corresponding control levels after 1 day or 3 days of recovery from 6 months of aestivation. This could help to restore the ability of *P*. *annectens* to excrete ammonia through the gills upon arousal.

## Conclusion

Our results reveal how aestivating *P*. *annectens* regulates the gene and protein expression levels of *rhag*/Rhag, *rhbg*/Rhbg and *rhcg*/Rhcg in its gills to impede ammonia excretion during the induction and maintenance phases and to regain the ability of ammonia excretion during the arousal phase. These results reiterate that aestivation involves functional modifications of the gills and the interplay between up-regulation and down-regulation of *rhgp*/Rhgp during the three phases of aestivation. At present, how the branchial epithelial cells avoided cell death during the maintenance phase and how they initiate the recovery process in response to the availability of water during the arousal phase remain enigmatic and await future studies.

## Supporting information

S1 Table Primers used for PCR, RACE and qPCR of *rhesus blood group-associated glycoprotein* (*rhag*), *rhesus family B glycoprotein* (*rhbg*) and *rhesus family C glycoprotein* (*rhcg*) from the gills of *Protopterus annectens*. (DOCX)

S2 Table List of selected species and their accession numbers used for dendrogram analyses of Rhag/RhAG. “*” indicates the outgroup.
(DOCX)

S3 Table List of selected species and their accession numbers used for dendrogram analyses of Rhbg/RhBG. “*” indicates the outgroup.
(DOCX)

S4 Table List of selected species and their accession numbers used for dendrogram analyses of Rhcg/RhCG. “*” indicates the outgroup.
(DOCX)

S5 Table The percentage similarity between the deduced amino acid sequence of Rhesus blood group-associated glycoprotein (Rhag) from *Protopterus annectens* and Rhag/RhAG from other animal species obtained from GenBank (accession numbers in brackets). (DOCX)

S6 Table The percentage similarity between the deduced amino acid sequence of Rhesus family B glycoprotein (Rhbg) from *Protopterus annectens* and Rhbg/RhBG from other animal species obtained from GenBank (accession numbers in brackets). (DOCX)

S7 Table The percentage similarity between the deduced amino acid sequence of Rhesus family C glycoprotein (Rhcg) from *Protopterus annectens* and Rhcg/RhCG from other animal species obtained from GenBank (accession numbers in brackets). (DOCX)
