# Supplementary material for: Molecular characterization of three Rhesus glycoproteins from the gills of the African lungfish, Protopterus annectens, and effects of aestivation on their mRNA expression levels and protein abundance
Source: PLoS One. 2017 Oct 26;12(10):e0185814. doi: 10.1371/journal.pone.0185814 (PMC5657625; doi:10.1371/journal.pone.0185814)
Supplement: S1 Table — (DOCX) [file pone.0185814.s001.docx]

**S1 Table. Primers used for PCR, RACE and qPCR of *rhesus blood group-associated glycoprotein* (*rhag*), *rhesus family B glycoprotein* (*rhbg*) and *rhesus family C glycoprotein* (*rhcg*) from the gills of *Protopterus annectens*.**

| **Gene** | **Primer type** | **Primer sequence (5’to 3’)** |
| --- | --- | --- |
| *rhag* | PCR | Forward: GCTATGAAACAAATGATGGTGG |
|  |  | Reverse: CGTGTGCCTCTTCTTCCT |
|  | 5’-RACE | AGACCCTTCATTATCGTGACCATTCCT |
|  | 3’-RACE | CAGCACAGCAACAGTTCTGATCTCCT |
|  | qPCR | Forward: CTCAGGAAACAACAGCGT |
|  |  | Reverse: CCAAAGCAGCAATAAGCA |
|  | PCR (IVT) | Forward: GATGATAATATGGCCACCACCCATATGCCTTCACATTATACA |
|  |  | Reverse: TTTTTTTTTTTTTTTTTTTTTCTATAGTTAGCTTGTGTTCTC |
| *rhbg* | PCR | Forward: ATGACTTCTACTTCCGCTATCC |
|  |  | Reverse: GGTTGATGCTGTCCTCTC |
|  | 5’-RACE | TCATCTCCATGGATAGTAACGGCTGAG |
|  | 3’-RACE | ACCTTTGGAGCATACTTTGGACTGACTG |
|  | qPCR | Forward: GGCATCAATCAGATAATTTCCC |
|  |  | Reverse: TGACTGACATCATCGTTCCA |
|  | PCR (IVT) | Forward: GATGATAATATGGCCACCACCCATATGACAGCTCACGCCACT |
|  |  | Reverse: TTTTTTTTTTTTTTTTTTTTTCTACAATAAGACAGGTGAAGG |
| *rhcg* | PCR | Forward: CTTCAGATACCCAAGTTTCCA |
|  |  | Reverse: ACCAACTAGAGTTCCTCCA |
|  | 5’-RACE | GCAGCAAGGGAGCAGTAAGTGTTAATGG |
|  | 3’-RACE | GGTGCTTCGTTTGGACTGATGGTATCGT |
|  | qPCR | Forward: GCCCTTGATATGTTTCATCTG |
|  |  | Reverse: CGTGGTGATACTATGATTCCT |
|  | PCR (IVT) | Forward: GATGATAATATGGCCACCACCCATATGGTGAACACAAATATG |
|  |  | Reverse: TTTTTTTTTTTTTTTTTTTTTCTATCAGTGTTTTGTATCTGT |

IVT, *in vitro* transcription and translation for the synthesis of the recombinant proteins of Rhag, Rhbg and Rhcg of *P. annectens*.
