# Supplementary material for: Molecular characterization of three Rhesus glycoproteins from the gills of the African lungfish, Protopterus annectens, and effects of aestivation on their mRNA expression levels and protein abundance
Source: PLoS One. 2017 Oct 26;12(10):e0185814. doi: 10.1371/journal.pone.0185814 (PMC5657625; doi:10.1371/journal.pone.0185814)
Supplement: S2 Table — “*” indicates the outgroup. (DOCX) [file pone.0185814.s002.docx]

**S2 Table.** A l**ist of selected species and their accession numbers used for dendrogram analyses of Rhag/RhAG.** “*” indicates the outgroup.

| **Species** | **Accession number** |
| --- | --- |
| *Alcolapia grahami* Rhag | AFZ78444.1 |
| *Anabas testudineus* Rhag | AIC81181.1 |
| *Bos taurus* RHAG | NP_776596.1 |
| *Canis lupus familiaris* RhAG | AAX39719.1 |
| *Cyprinus carpio* Rhag | AGN71674.1 |
| *Danio rerio* Rhag | AAQ10011.1 |
| *Gasterosteus aculeatus* Rhag | ABF69688.1 |
| *Homo sapiens* RhAG | NP_000315.2 |
| *Macaca mulatta* RhAG | NP_001027987.1 |
| *Mus musculus* RhAG | AAI01942.1 |
| *Oncorhynchus mykiss* Rhag | ABV24962.1 |
| *Opsanus beta* Rhag | AEA77167.1 |
| *Pan troglodytes* RhAG | NP_001009033.1 |
| *Porichthys notatus* Rhag | AGA93878.1 |
| *Rattus norvegicus* RhAG | EDM18672.1 |
| *Sus scrofa* RhAG | XP_003128488.1 |
| *Xenopus (Silurana) tropicalis* Rhag | XP_002933645.2 |
| *Xenopus laevis* Rhag | BAB13345.1 |
| *Ciona intestinalis* Rhag* | AAP87368.1 |
