# Supplementary material for: Molecular characterization of three Rhesus glycoproteins from the gills of the African lungfish, Protopterus annectens, and effects of aestivation on their mRNA expression levels and protein abundance
Source: PLoS One. 2017 Oct 26;12(10):e0185814. doi: 10.1371/journal.pone.0185814 (PMC5657625; doi:10.1371/journal.pone.0185814)
Supplement: S3 Table — “*” indicates the outgroup. (DOCX) [file pone.0185814.s003.docx]

**S3 Table. A list of selected species and their accession numbers used for dendrogram analyses of Rhbg/RhBG.** “*” indicates the outgroup.

| **Species** | **Accession number** |
| --- | --- |
| *Alcolapia grahami* Rhbg | AFZ78445.1 |
| *Anabas testudineus* Rhbg | AIC81182.1 |
| *Callorhinchus milii* Rhbg | AFP03342.1 |
| *Canis lupus familiaris* RhBG | AAV40851.1 |
| *Cyprinus carpio* Rhbg | AHJ59465.1 |
| *Danio rerio* Rhbg | AAQ09527.1 |
| *Gasterosteus aculeatus* Rhbg | ABF69689.1 |
| *Homo sapiens* RhBGA | NP_065140.3 |
| *Homo sapiens* RhBGB | NP_001243324.1 |
| *Homo sapiens* RhBGC | NP_001243325.1 |
| *Larimichthys crocea* Rhbg | KKF24588.1 |
| *Mus musculus* RhBG | AAF19371.1 |
| *Oncorhynchus mykiss* Rhbg | NP_001118134.1 |
| *Opsanus beta* Rhbg | AEA77168.1 |
| *Oryzias latipes* Rhbg | NP_001098561.1 |
| *Porichthys notatus* Rhbg | AGA93879.1 |
| *Rattus norvegicus* RhBG | AAN07790.1 |
| *Squalus acanthias* Rhbg | AJF44128.1 |
| *Sus scrofa* RhBG | AAK14651.1 |
| *Takifugu rubripes* Rhbg | AAM48577.1 |
| *Tetraodon nigroviridis* Rhbg | AAY41906.1 |
| *Xenopus (Silurana) tropicalis* Rhbg | AAU89493.1 |
| *Xenopus laevis* Rhbga | NP_001083174.1 |
| *Xenopus laevis* Rhbgb | NP_001087152.1 |
| *Ciona intestinalis* Rhbg* | AAY41909.1 |
