# Supplementary material for: Molecular characterization of three Rhesus glycoproteins from the gills of the African lungfish, Protopterus annectens, and effects of aestivation on their mRNA expression levels and protein abundance
Source: PLoS One. 2017 Oct 26;12(10):e0185814. doi: 10.1371/journal.pone.0185814 (PMC5657625; doi:10.1371/journal.pone.0185814)
Supplement: S4 Table — “*” indicates the outgroup. (DOCX) [file pone.0185814.s004.docx]

**S4 Table. A list of selected species and their accession numbers used for dendrogram analyses of Rhcg/RhCG.** “*” indicates the outgroup.

| **Species** | **Accession number** |
| --- | --- |
| *Anabas testudineus* Rhcg1 | AIC81183.1 |
| *Anabas testudineus* Rhcg2 | AIC81184.1 |
| *Bos taurus* RhCG | AAK14650.1 |
| *Callorhinchus milii* Rhcg | AFO96383.1 |
| *Danio rerio* Rhcg1 | AAM90586.1 |
| *Danio rerio* Rhcg2a | BAF63791.1 |
| *Danio rerio* Rhcg2b | BAF63792.1 |
| *Gasterosteus aculeatus* Rhcg | ABF69690.1 |
| *Homo sapiens* RhCG | AAF19372.1 |
| *Ictalurus punctatus* Rhcg1 | AHH37525.1 |
| *Larimichthys crocea* Rhcg1 | KKF31984.1 |
| *Larimichthys crocea* Rhcg2 | KKF19632.1 |
| *Lipophrys pholis* Rhcg1a | AGU71416.1 |
| *Lipophrys pholis* Rhcg1b | AGU71417.1 |
| *Lipophrys pholis* Rhcg2 | AGU71418.1 |
| *Macaca mulatta* RhCG | ABD72472.1 |
| *Mus musculus* RhCG | AAF19373.1 |
| *Oncorhynchus mykiss* Rhcg | AAU89494.1 |
| *Oryzias latipes* Rhcg | XP_004069769.1 |
| *Pan troglodytes* RhCG | AAX39717.1 |
| *Rattus norvegicus* RhCG | AAN07791.1 |
| *Sus scrofa* RhCG | ABF69687.1 |
| *Tetraodon nigroviridis* Rhcg | AAY41907.1 |
| *Xenopus (Silurana) tropicalis* Rhcg | AAQ02688.1 |
| *Xenopus laevis* Rhcg | NP_001088553.1 |
| *Thelohanellus kitauei* Rhcg* | KII73745.1 |
