# Supplementary material for: Molecular characterization of three Rhesus glycoproteins from the gills of the African lungfish, Protopterus annectens, and effects of aestivation on their mRNA expression levels and protein abundance
Source: PLoS One. 2017 Oct 26;12(10):e0185814. doi: 10.1371/journal.pone.0185814 (PMC5657625; doi:10.1371/journal.pone.0185814)
Supplement: S5 Table — (DOCX) [file pone.0185814.s005.docx]

**S5 Table. The percentage similarity between the deduced amino acid sequence of Rhesus blood group-associated glycoprotein (Rhag) from *Protopterus annectens* and Rhag/RhAG from other animal species obtained from GenBank (accession numbers in parentheses).**

| **Classification** | **Species** | **Similarity** |
| --- | --- | --- |
| **Amphibians** | *Xenopus (Silurana) tropicalis* Rhag (XP_002933645.2) | 73.1% |
|  | *Xenopus laevis* Rhag (BAB13345.1) | 70.1% |
| **Actinopterygians** | *Oncorhynchus mykiss* Rhag (ABV24962.1) | 71.7% |
|  | *Gasterosteus aculeatus* Rhag (ABF69688.1) | 71.4% |
|  | *Alcolapia grahami* Rhag (AFZ78444.1) | 70.8% |
|  | *Danio rerio* Rhag (AAQ10011.1) | 69.9% |
|  | *Anabas testudineus* Rhag (AIC81181.1) | 69.8% |
|  | *Cyprinus carpio* Rhag (AGN71674.1) | 69.4% |
|  | *Opsanus beta* Rhag (AEA77167.1) | 69.1% |
|  | *Porichthys notatus* Rhag (AGA93878.1) | 69.1% |
| **Mammals** | *Macaca mulatta* RhAG (NP_001027987.1) | 64.9% |
|  | *Pan troglodytes* RhAG (NP_001009033.1) | 64.0% |
|  | *Homo sapiens* RhAG (NP_000315.2) | 63.5% |
|  | *Canis lupus familiaris* RhAG (AAX39719.1) | 62.5% |
|  | *Mus musculus* RhAG (AAI01942.1) | 62.4% |
|  | *Rattus norvegicus* RhAG (EDM18672.1) | 60.3% |
|  | *Bos taurus* RhAG (NP_776596.1) | 59.9% |
|  | *Sus scrofa* RhAG (XP_003128488.1) | 59.6% |

Sequences are arranged in a descending order of similarity.

**S6 Table. The percentage similarity between the deduced amino acid sequence of Rhesus family B glycoprotein (Rhbg) from *Protopterus annectens* and Rhbg/RhBG from other animal species obtained from GenBank (accession numbers in parentheses).**

| **Classification** | **Species** | **Similarity** |
| --- | --- | --- |
| **Chondrichthyes** | *Squalus acanthias* Rhbg (AJF44128.1) | 69.2% |
|  | *Callorhinchus milii* Rhbg (AFP03342.1) | 64.9% |
| **Actinopterygians** | *Alcolapia grahami* Rhbg (AFZ78445.1) | 69.0% |
|  | *Oncorhynchus mykiss* Rhbg (NP_001118134.1) | 68.9% |
|  | *Opsanus beta* Rhbg (AEA77168.1) | 68.6% |
|  | *Anabas testudineus* Rhbg (AIC81182.1) | 68.4% |
|  | *Porichthys notatus* Rhbg (AGA93879.1) | 68.4% |
|  | *Larimichthys crocea* Rhbg (KKF24588.1) | 67.5% |
|  | *Oryzias latipes* Rhbg (NP_001098561.1) | 67.3% |
|  | *Tetraodon nigroviridis* Rhbg (AAY41906.1) | 66.5% |
|  | *Gasterosteus aculeatus* Rhbg (ABF69689.1) | 65.5% |
|  | *Cyprinus carpio* Rhbg (AHJ59465.1) | 65.0% |
|  | *Danio rerio* Rhbg (AAQ09527.1) | 64.2% |
|  | *Takifugu rubripes* Rhbg (AAM48577.1) | 63.5% |
| **Amphibians** | *Xenopus laevis* Rhbgb (NP_001087152.1) | 68.2% |
|  | *Xenopus laevis* Rhbga (NP_001083174.1) | 67.8% |
|  | *Xenopus (Silurana) tropicalis* Rhbg (AAU89493.1) | 66.4% |
| **Mammals** | *Sus scrofa* RhBG (AAK14651.1) | 58.1% |
|  | *Canis lupus familiaris* RhBG (AAV40851.1) | 58.1% |
|  | *Rattus norvegicus* RhBG (AAN07790.1) | 57.4% |
|  | *Mus musculus* RhBG (AAF19371.1) | 57.0% |
|  | *Homo sapiens* RhBGA (NP_065140.3) | 56.6% |
|  | *Homo sapiens* RhBGC (NP_001243325.1) | 51.1% |
|  | *Homo sapiens* RhBGB (NP_001243324.1) | 49.4% |

Sequences are arranged in a descending order of similarity.

**S7 Table. The percentage similarity between the deduced amino acid sequence of Rhesus family C glycoprotein (Rhcg) from *Protopterus annectens* and Rhcg/RhCG from other animal species obtained from GenBank (accession numbers in parentheses).**

| **Classification** | **Species** | **Similarity** |
| --- | --- | --- |
| **Actinopterygians** | *Tetraodon nigroviridis* Rhcg (AAY41907.1) | 65.7% |
|  | *Lipophrys pholis* Rhcg1a (AGU71416.1) | 64.0% |
|  | *Lipophrys pholis* Rhcg1b (AGU71417.1) | 64.0% |
|  | *Ictalurus punctatus* Rhcg1 (AHH37525.1) | 64.0% |
|  | *Anabas testudineus* Rhcg2 (AIC81184.1) | 63.5% |
|  | *Larimichthys crocea* Rhcg1 (KKF31984.1) | 63.3% |
|  | *Danio rerio* Rhcg2a (BAF63791.1) | 63.2% |
|  | *Anabas testudineus* Rhcg1 (AIC81183.1) | 62.7% |
|  | *Danio rerio* Rhcg2b (BAF63792.1) | 62.6% |
|  | *Larimichthys crocea* Rhcg2 (KKF19632.1) | 62.5% |
|  | *Oncorhynchus mykiss* Rhcg (AAU89494.1) | 61.8% |
|  | *Danio rerio* Rhcg1 (AAM90586.1) | 61.8% |
|  | *Gasterosteus aculeatus* Rhcg (ABF69690.1) | 61.3% |
|  | *Lipophrys pholis* Rhcg2 (AGU71418.1) | 60.9% |
|  | *Oryzias latipes* Rhcg (XP_004069769.1) | 60.2% |
| **Chondrichthyes** | *Callorhinchus milii* Rhcg (AFO96383.1) | 64.2% |
| **Amphibians** | *Xenopus laevis* Rhcg (NP_001088553.1) | 64.1% |
|  | *Xenopus (Silurana) tropicalis* Rhcg (AAQ02688.1) | 63.4% |
| **Mammals** | *Sus scrofa* RhCG (ABF69687.1) | 61.5% |
|  | *Homo sapiens* RhCG (AAF19372.1) | 60.3% |
|  | *Pan troglodytes* RhCG (AAX39717.1) | 60.3% |
|  | *Macaca mulatta* RhCG (ABD72472.1) | 59.7% |
|  | *Bos taurus* RhCG (AAK14650.1) | 58.7% |
|  | *Mus musculus* RhCG (AAF19373.1) | 55.5% |
|  | *Rattus norvegicus* RhCG (AAN07791.1) | 55.4% |

Sequences are arranged in a descending order of similarity.
