# Supplementary material for: Molecular characterization of three Rhesus glycoproteins from the gills of the African lungfish, Protopterus annectens, and effects of aestivation on their mRNA expression levels and protein abundance
Source: PLoS One. 2017 Oct 26;12(10):e0185814. doi: 10.1371/journal.pone.0185814 (PMC5657625; doi:10.1371/journal.pone.0185814)
Supplement: S6 Table — (DOCX) [file pone.0185814.s006.docx]

**S6 Table. The percentage similarity between the deduced amino acid sequence of Rhesus family B glycoprotein (Rhbg) from *Protopterus annectens* and Rhbg/RhBG from other animal species obtained from GenBank (accession numbers in parentheses).**

| **Classification** | **Species** | **Similarity** |
| --- | --- | --- |
| **Chondrichthyes** | *Squalus acanthias* Rhbg (AJF44128.1) | 69.2% |
|  | *Callorhinchus milii* Rhbg (AFP03342.1) | 64.9% |
| **Actinopterygians** | *Alcolapia grahami* Rhbg (AFZ78445.1) | 69.0% |
|  | *Oncorhynchus mykiss* Rhbg (NP_001118134.1) | 68.9% |
|  | *Opsanus beta* Rhbg (AEA77168.1) | 68.6% |
|  | *Anabas testudineus* Rhbg (AIC81182.1) | 68.4% |
|  | *Porichthys notatus* Rhbg (AGA93879.1) | 68.4% |
|  | *Larimichthys crocea* Rhbg (KKF24588.1) | 67.5% |
|  | *Oryzias latipes* Rhbg (NP_001098561.1) | 67.3% |
|  | *Tetraodon nigroviridis* Rhbg (AAY41906.1) | 66.5% |
|  | *Gasterosteus aculeatus* Rhbg (ABF69689.1) | 65.5% |
|  | *Cyprinus carpio* Rhbg (AHJ59465.1) | 65.0% |
|  | *Danio rerio* Rhbg (AAQ09527.1) | 64.2% |
|  | *Takifugu rubripes* Rhbg (AAM48577.1) | 63.5% |
| **Amphibians** | *Xenopus laevis* Rhbgb (NP_001087152.1) | 68.2% |
|  | *Xenopus laevis* Rhbga (NP_001083174.1) | 67.8% |
|  | *Xenopus (Silurana) tropicalis* Rhbg (AAU89493.1) | 66.4% |
| **Mammals** | *Sus scrofa* RhBG (AAK14651.1) | 58.1% |
|  | *Canis lupus familiaris* RhBG (AAV40851.1) | 58.1% |
|  | *Rattus norvegicus* RhBG (AAN07790.1) | 57.4% |
|  | *Mus musculus* RhBG (AAF19371.1) | 57.0% |
|  | *Homo sapiens* RhBGA (NP_065140.3) | 56.6% |
|  | *Homo sapiens* RhBGC (NP_001243325.1) | 51.1% |
|  | *Homo sapiens* RhBGB (NP_001243324.1) | 49.4% |

Sequences are arranged in a descending order of similarity.
