# Supplementary material for: Molecular characterization of three Rhesus glycoproteins from the gills of the African lungfish, Protopterus annectens, and effects of aestivation on their mRNA expression levels and protein abundance
Source: PLoS One. 2017 Oct 26;12(10):e0185814. doi: 10.1371/journal.pone.0185814 (PMC5657625; doi:10.1371/journal.pone.0185814)
Supplement: S7 Table — (DOCX) [file pone.0185814.s007.docx]

**S7 Table. The percentage similarity between the deduced amino acid sequence of Rhesus family C glycoprotein (Rhcg) from *Protopterus annectens* and Rhcg/RhCG from other animal species obtained from GenBank (accession numbers in parentheses).**

| **Classification** | **Species** | **Similarity** |
| --- | --- | --- |
| **Actinopterygians** | *Tetraodon nigroviridis* Rhcg (AAY41907.1) | 65.7% |
|  | *Lipophrys pholis* Rhcg1a (AGU71416.1) | 64.0% |
|  | *Lipophrys pholis* Rhcg1b (AGU71417.1) | 64.0% |
|  | *Ictalurus punctatus* Rhcg1 (AHH37525.1) | 64.0% |
|  | *Anabas testudineus* Rhcg2 (AIC81184.1) | 63.5% |
|  | *Larimichthys crocea* Rhcg1 (KKF31984.1) | 63.3% |
|  | *Danio rerio* Rhcg2a (BAF63791.1) | 63.2% |
|  | *Anabas testudineus* Rhcg1 (AIC81183.1) | 62.7% |
|  | *Danio rerio* Rhcg2b (BAF63792.1) | 62.6% |
|  | *Larimichthys crocea* Rhcg2 (KKF19632.1) | 62.5% |
|  | *Oncorhynchus mykiss* Rhcg (AAU89494.1) | 61.8% |
|  | *Danio rerio* Rhcg1 (AAM90586.1) | 61.8% |
|  | *Gasterosteus aculeatus* Rhcg (ABF69690.1) | 61.3% |
|  | *Lipophrys pholis* Rhcg2 (AGU71418.1) | 60.9% |
|  | *Oryzias latipes* Rhcg (XP_004069769.1) | 60.2% |
| **Chondrichthyes** | *Callorhinchus milii* Rhcg (AFO96383.1) | 64.2% |
| **Amphibians** | *Xenopus laevis* Rhcg (NP_001088553.1) | 64.1% |
|  | *Xenopus (Silurana) tropicalis* Rhcg (AAQ02688.1) | 63.4% |
| **Mammals** | *Sus scrofa* RhCG (ABF69687.1) | 61.5% |
|  | *Homo sapiens* RhCG (AAF19372.1) | 60.3% |
|  | *Pan troglodytes* RhCG (AAX39717.1) | 60.3% |
|  | *Macaca mulatta* RhCG (ABD72472.1) | 59.7% |
|  | *Bos taurus* RhCG (AAK14650.1) | 58.7% |
|  | *Mus musculus* RhCG (AAF19373.1) | 55.5% |
|  | *Rattus norvegicus* RhCG (AAN07791.1) | 55.4% |

Sequences are arranged in a descending order of similarity.
